# Ectonucleotidases as bridge between the ATP and adenosine world: reflections on Geoffrey Burnstock

**DOI:** 10.1007/s11302-022-09862-6

**Published:** 2022-05-06

**Authors:** Jürgen Schrader

**Affiliations:** Department of Molecular Cardiology, University of Düsseldorf, Medical Faculty, Universitaetsstr. 1, 40225 Duesseldorf, Germany

**Keywords:** ATP, Adenosine, Ectoenzymes, CD73, Metabolism, Burnstock

## Abstract

Historically, mainly by the work of Robert Berne, extracellular adenosine was the first purine compound recognized as an important signaling molecule linking energy metabolism to function by acting on membrane bound receptors. Geoffrey Burnstock by his vision and endurance pioneered the idea that cells release ATP that also acts as an extracellular signaling molecule under many physiological and pathophysiological circumstances. Only later, it was appreciated that extracellular ATP and adenosine are metabolically linked by the activity of several ectoenzymes which critically determine the concentrations of these purines at their respective receptors. In this brief review, I will report some personal recollections on Geoffrey Burnstock and his impressive personality. In addition, I will give a brief overview on our present knowledge of extracellular purine metabolism and its control and will address some still open issues.

## Introduction

Jeoffrey Burnstock (1929–2020) entered my scientific live very early in my career. After a postdoc with Robert M. Berne (1918–2001) in Charlottesville in the early 1970s, — Berne’s lab was leading adenosine research in these times — I came across Jeoff’s now famous 1972 review in Pharmacological Reviews on Purinergic Nerves, proposing a central role for extracellular ATP [1]. I still remember that even after careful reading, I found Jeoff’s proposal not fully convincing, since the evidence was indirect and released ATP has not been directly measured. The adenosine field at that time was much more advanced and there were analytical techniques available to quantify this nucleoside and link its concentration to function. Reliably measuring ATP has been a major task in the years to follow since it was and still is difficult to differentiate analytically between lytic ATP release from dying cells and specific ATP release mechanisms. In addition, Berne’s adenosine hypothesis for metabolic coronary flow regulation at that time assumed that most of the adenosine during hypoxia is formed within the cell and then diffuses into extracellular space to become functionally active. The history of adenosine research and Berne’s contribution was elegantly reviewed by Ray Olsson [2]. At that time, there was conceptually no need for extracellular ATP being a precursor of adenosine at least in the cardiovascular field.

This perspective fundamentally changed in the 1990s when it was discovered, mainly by the work of Herbert Zimmermann and Gennady Yegutkin, that there are various ectoenzymes present at the outer cell surface which can effectively break down extracellular ATP to adenosine [3, 4]. This pathway did not require hypoxia, but was it also functionally important? Knockout of CD39 by the Robson lab [5] and CD73 in my [6] and Linda Thompson’s lab [7] clearly showed that the ectoenzyme pathway was indeed quantitatively important and resulted in major cardiovascular phenotypes under stressed conditions. Metabolically speaking, there must have been a continuous cellular release of ATP which provides AMP, the substrate of CD73. Jeoffrey Burnstock was proven right!

## How it all began

**Fig. 1:**
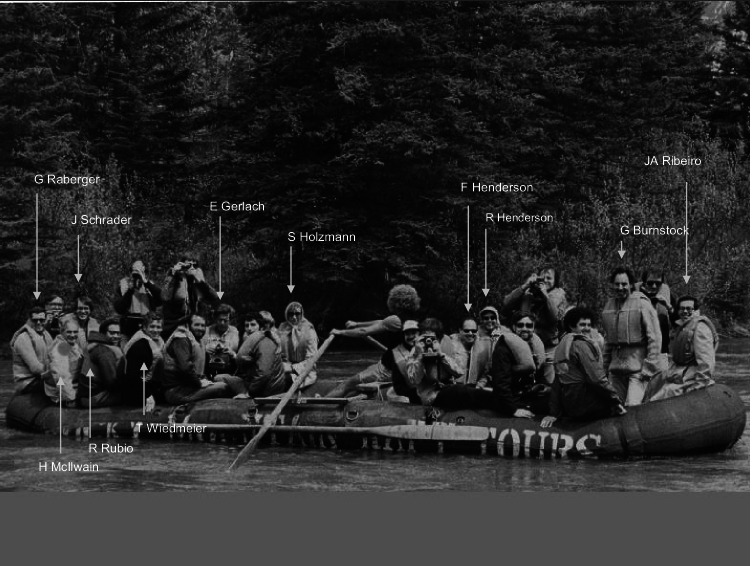
Boat trip in Banff, Canada, during the first conference on “Physiological and Regulatory Functions of Adenosine and Adenine Nucleotides” (1979). Geoff Burnstock is standing on the right side of the boat accompanied by Ruth and Frank Henderson, Edmonton, and Alexandre Ribeiro, Lisbon. The adenosine group on the left comprises Tom Wiedmeier, Charlottesville (Berne lab), Raphael Rubio, Charlottesville; Henry McIlwain, London; Gerhard Raberger, Vienna; Jürgen Schrader, Munich at that time; Eckerhard Gerlach, Munich, and Sigrid Holzmann, Graz, Austria

The first of a series of conferences which brought the ATP and adenosine world together was organized by Hans Baer and George Drummond in 1978 in Banff, Canada, and was entitled “Physiologic and regulatory functions of adenosine and adenine nucleotides” [8]. This meeting was intellectually exiting and marked the point in time when the field took off. Great outdoor activities facilitated the exchange ideas and getting to know the participants coming from chemistry, pharmacology, and physiology. Figure 1 shows some of the participants of the Banff conference on a boat trip. Interestingly, the ATP group with Jeoff Burnstock standing up was sitting on the front side of the boat, while the adenosine group occupied the other side. This somewhat reflects that historically, there used to be — for quite some time — a distinct ATP world and adenosine world, and the proponents of each acted sometimes like enemy brothers, on meetings and otherwise.

In a broader view, however, the two systems are part of an exiting regulatory network in living tissue that is connected by ectoenzymes. It soon became obvious that it is the enzymatic activity of these ectoenzymes that critically determines whether cells of a given tissue are exposed to a predominant ATP or adenosine environment. Clearly, extracellular purine metabolism is very dynamic and is controlled by many variables. With extracellular ATP and adenosine enzymatically interconnected, nature has developed a complex but highly interesting regulatory system which we are only beginning to understand.

## Jeoff the mover

Jeoff certainly was a man with great vision and particularly his enthusiasm for purinergic signaling was contagious and has stimulated many talented researches to enter the field. For example, he collaborated with Eric Barnard (1927–2018), an eminent molecular biologist in London, and they both were the first to characterize and clone G-protein-coupled receptor for ATP [9]. Since there are receptors for ATP, there must be extracellular ATP and this made the ATP signaling circle complete. Jeoff’s original hypothesis was now on firm molecular grounds.

Jeoff’s ability to infuse enthusiasm was with him from his early school time. In a personal reflection on his live during one of his last plenary lectures, he told the story that during his time at school, he was very keen on table tennis. After a while he managed that not only his class but also the entire school was involved in regular table tennis tournaments. This was only put to an end, after the director of the school declared that there are other important sports aside of table tennis.

One of Geoff’s strength, as I remember him, was his ability to integrate the many publications in the purinergic signaling field into a coherent picture. He wanted to understand how purines in a living system really worked and whether one could therapeutically interfere. To this end, he knew the relevant literature very well and was a master in presenting it! I still remember receiving — in the pre-electronic area — regular postcards from him with request for a reprint. Geoff wrote many excellent reviews. Well cited is his review on “Receptors for purines and pyrimidines” [10] with over 1 000 references. His last review on the therapeutic developments in purinergic signaling, published in 2017, is another impressive example in this respect [11]. With more than 900 references, he covers the potential relevance of purinergic signaling in all disease states so far investigated. This paper provides important insights and will be a valuable literature resource for the future.

## Where do we presently stand?

**Fig. 2:**
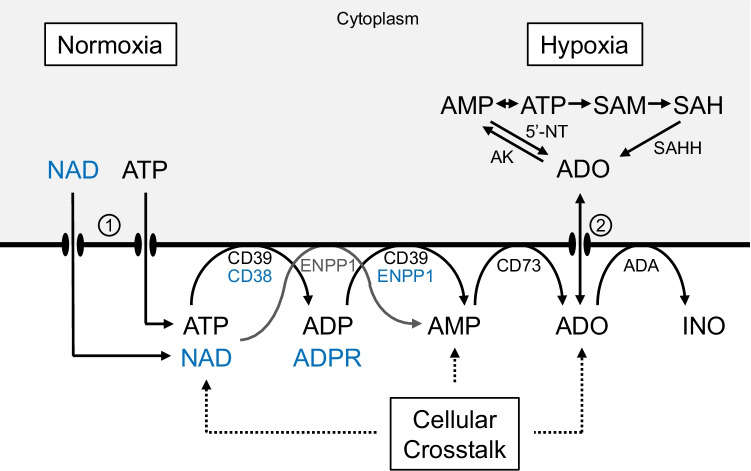
Schematic outline of the main extracellular and intracellular pathways catalyzing the breakdown of ATP/NAD to adenosine. During normoxic conditions, most cells within a tissue release ATP and NAD into the interstitial space, particularly when stimulated. This involves various transmembrane transporters. Adenosine formed by CD73 can be removed by cellular reuptake (equilibrative and concentrative nucleoside transporter proteins ENTs and CNTs, respectively) and deamination to inosine (ADA = ecto-adenosine deaminase bound to CD26) and further to hypoxanthin. Note, that aside of CD39, ATP can be directly degraded to AMP by action of ectonucleotide pyrophosphatase/phosphodiesterase I (ENPP1). CD38 is transmembrane glycoprotein that hydrolyzes extracellular NAD to adenosine diphosphate ribose (ADPR) which is further converted to AMP by ENPP1 (alternative adenosine forming pathway). During tissue hypoxia, intracellular adenosine becomes strongly elevated by the concerted action of elevated substrate AMP levels and inhibition of adenosine kinase (AK) which interferes with the “futile” metabolic cycle between AMP and adenosine involving cytosolic 5′-ncleotidase (5′-NT). Adenosine can also be formed by the cellular transmethylation pathway [S-adenosyl-methionine (SAM) → S-adenosyl-homocysteine (SAH) involving SAH-hydrolase (SAHH)]; however, the input of this pathway to adenosine formation is generally only small. Cellular crosstalk refers to the fact that within an intact tissue, the extracellular concentration of various purine compounds is finally determined by the release of ATP and ectoenzyme activities of all cell types, which form the interstitial fluid space in a living tissue

We now know that ATP and adenosine are metabolically well interconnected by several ectonucleotidases (Fig. 2) which play an important role in purinergic signaling by P1 and P2 receptors. Several recent reviews competently summarize our present knowledge on the individual enzymes involved [12, 13], their role in acute and chronic inflammation [14] in human diseases [15], including history of ectonucleotidases [16], and potential checkpoint inhibitors in cancer treatment [17].

Figure 2 depicts our present view on the major ectoenzymes involved and corresponding metabolites which constitutes a metabolic pathway. In the following, I will briefly address some general regulatory principles of metabolic control which also apply to this extracellular pathway from ATP to adenosine. The conceptional basis for regulation in metabolic pathways was laid by the Oxford biochemist Eric Arthur Newsholme (1935–2011) [18] and was more recently applied to state-of-the-art studies [19]. By using thermodynamic concepts, Newsholme postulated that accurate measurements of the concentrations of the intermediary substrates and products as well as product/substrate ratios are required to characterize the nature of the reaction catalyzed by each enzyme. Such measurements are feasible in isolated cells in culture but are difficult in intact tissue since the assessment of the composition of the extracellular fluid space is technically demanding. In the case of ATP, Di Virgilio and colleagues [20] have elegantly engineered a chimeric plasma membrane luciferase (pmeLUC), that is targeted to the outer plasma membrane and permits sensitive interstitial ATP measurements [20]. Comparable sensors for adenosine are presently not available and at best rely on electrochemical microsensors [21, 22] and microdialysis [23]. In the future, it would be highly desirable to be able to non-invasively quantify extracellular ATP, ADP, and adenosine in a complex tissue context, since it is finally the concentration of these purine compounds which elicits signaling at the respective P1 and P2 receptors.

Each metabolic pathway starts with a flux-generating step and ends with the loss of the products or may constitute the start of another metabolic pathway. Cleary, the ectonucleotidase cascade is initiated by the cellular release ATP and NAD by various mechanisms [24]. The actual purine concentration is determined by the rate of ATP and NAD degradation. Similar consideration apply to adenosine, the concentration of which under normoxia is critically determined by two kinetic parameters: rate of production from AMP as compared to the rate of adenosine removal by reuptake into the cell (salvage pathway) [25] and deamination to inosine [26]. Aside of the activity of CD73, also the substrate concentration of AMP matters. Theoretically, it is possible that despite increased CD73 activity, this might go along with unchanged or even decreased adenosine levels, whenever the rate of deamination and cellular reuptake is equal or exceeds adenosine production! Whether such a metabolic situation is biologically realized is presently not known.

From these considerations, it is evident that it may be misleading to extrapolate — as frequently done — from measurements of enhanced CD73 at the gene or protein level to elevated adenosine levels in respective tissues. This the more, since CD73 activity can be inhibited by NAD-ribosylation [27] which can explain the profound inhibition of CD73-dependent formation of anti-inflammatory adenosine in B cells of SLE patients recently reported by us [28, 29]. Again, this calls for reliable measurements of ATP and adenosine in the interstitial space of organs and tumors.

Under normoxic conditions, most of the extracellular adenosine is derived by the concerted action of the various ectonucleotidases such as CD39, CD38, and ENNP1 (Fig. 2). In tumors, ENPP1 was recently shown to also selectively degrade extracellular cGAMP, an immune-stimulatory metabolite whose breakdown product is adenosine [30]. The extracellular adenosine concentration normally exceeds intracellular adenosine so that the flux is from outside to inside, parenchymal cell representing a sink, not a source, for adenosine [31]. Under hypoxic conditions, however, the situation dramatically changes in that the gradient is reversed and adenosine accumulates in a PO2-dependent manner [32]. The metabolic basis for this effect is the existence of an intracellular “futile cycle” between AMP and adenosine involving cytosolic 5′-nucleotidase and adenosine kinase. Substrate cycles as efficient mechanisms to improve control sensitivity have already been discussed by Newsholme [18]. The biological function of this high turnover AMP-adenosine metabolic cycle is that inhibition of adenosine kinase activity can substantially augment intracellular adenosine thereby steepening the gradient to the extracellular space. Interestingly, hypoxia induces inhibition of adenosine kinase whereby small changes in hypoxia-induced free AMP are converted into a major rise in adenosine [33]. Since the metabolic cycle between AMP and adenosine is linked to the transmethylation pathway from SAM to SAH (Fig. 2), it is not surprising that adenosine kinase appears also to be important in determining the global methylation status of DNA, thereby contributing to epigenomic regulation [34]. Taken together, extracellular adenosine formed by hypoxia is most likely derived from both the extracellular degradation of ATP and the direct cellular export by nucleoside transporters. Use of highly specific ecto-ATPase inhibitor can differentiate between these two possibilities [35]. Inhibition of adenosine kinase is still an elegant means to augment adenosine under normoxic conditions.

## What comes next?

Given the complexities of extracellular purine metabolism, future studies on the dynamics of this pathway might involve the application of isotopically labelled tracers combined with NMR and mass spectrometry, techniques that were successfully applied in the past to metabolic pathways in various organs [36]. Challenges persist as to the accurate estimation of substrate concentrations and flux rates from stable-isotope tracers. While the principles of metabolic regulation are similar, organ/cell-specific differences are likely to exist and need to be considered. With the advent of single cell transciptomics and spatial proteomics, we have now to integrate the ATP release from various cellular sources which finally determine in an intact tissue the interstitial purine concentration to which the ATP and adenosine receptors are exposed to. A better understanding on the dynamics of extracellular ATP degradation to adenosine in vivo will certainly provide a rational basis for the development of novel therapeutic strategies.

## Data Availability

Not applicable.
